# Low Cholesterol associated with TB in people living with HIV in an Asia-Pacific cohort

**DOI:** 10.1097/QAI.0000000000003761

**Published:** 2026-01-01

**Authors:** Rebecca T Henry, Vohith Khol, Cuong Duy Do, Ivan Marbaniang, I Ketut Agus Somia, Nagalingeswaran Kumarasamy, Evy Yunihastuti, Iskandar Azwa, Rossana Ditangco, Sasisopin Kiertiburanakul, Man Po Lee, Anchalee Avihingsanon, Hsin Pai Chen, Romanee Chaiwarith, Suwimon Khusuwan, Thach Ngoc, Sanjay Pujari, Chiaw Yee Choy, Jun Yong Choi, Yasmin Gani, Haruka Uemura, Jeremy Ross, Awachana Jiamsakul

**Affiliations:** 1 The Kirby Institute, UNSW Sydney, Australia; 2 National Center for HIV/AIDS, Dermatology & STDs, Phnom Penh, Cambodia; 3 Bach Mai Hospital, Hanoi, Vietnam; 4 BJ Government Medical College and Sassoon General Hospital, Pune, India; 5 Faculty of Medicine Udayana University - Ngoerah Hospital, Bali, Indonesia; 6 VHS-Infectious Diseases Medical Centre, Chennai Antiviral Research and Treatment Clinical Research Site (CART CRS), Voluntary Health Services, Chennai, India; 7 Faculty of Medicine Universitas Indonesia – Dr. Cipto Mangunkusumo General Hospital, Jakarta, Indonesia; 8 University Malaya Medical Centre, Kuala Lumpur, Malaysia; 9 Research Institute for Tropical Medicine, Muntinlupa City, Philippines; 10 Faculty of Medicine Ramathibodi Hospital, Mahidol University, Bangkok, Thailand; 11 Queen Elizabeth Hospital, Hong Kong SAR; 12 HIV-NAT/Thai Red Cross AIDS and Infectious Diseases Research Centre and Center of Excellence in Tuberculosis, Faculty of Medicine, Chulalongkorn University, Bangkok, Thailand; 13 Taipei Veterans General Hospital, Taipei, Taiwan; 14 Faculty of Medicine and Research Institute for Health Sciences, Chiang Mai University, Chiang Mai, Thailand; 15 Chiangrai Prachanukroh Hospital, Chiang Rai, Thailand; 16 National Hospital for Tropical Diseases, Hanoi, Vietnam; 17 Institute of Infectious Diseases, Pune, India; 18 Tan Tock Seng Hospital, National Centre for Infectious Diseases, Singapore; 19 Division of Infectious Diseases, Department of Internal Medicine, Yonsei University College of Medicine, Seoul, South Korea; 20 Hospital Sungai Buloh, Sungai Buloh, Malaysia; 21 National Center for Global Health and Medicine, Tokyo, Japan; 22 TREAT Asia, amfAR - The Foundation for AIDS Research, Bangkok, Thailand

**Keywords:** Tuberculosis, Human Immunodeficiency Virus, Cholesterol, CD4 Lymphocyte Count, Case-Control Studies, Odds Ratio

## Abstract

**Background:**

Tuberculosis (TB) remains the leading cause of illness and death among people living with HIV (PLHIV), particularly in high-burden areas. This study examined associations between TB and routine clinical markers: serum cholesterol, platelet count, and CD4 cell count.

**Setting:**

The analysis included data from the TREAT Asia HIV Observational Database (TAHOD), a multicenter cohort of adult PLHIV receiving care across the Asia-Pacific region.

**Methods:**

We conducted a cross-sectional matched case–control study of prospective and retrospective TB cases, comparing clinical and laboratory data within ±3 months of TB diagnosis. Conditional logistic regression assessed associations between TB and covariates.

**Results:**

The analysis included 4,244 PLHIV from 20 sites: 1,427 TB cases and 2,817 matched controls. TB cases were predominantly male (75.3%) and 45.7% aged 31–40. Multivariable analysis showed greater odds of TB diagnosis among males, those with low BMI, prior AIDS diagnosis, high HIV viral load, low CD4+ counts, or low total cholesterol. CD4+ counts <200 cells/μL had higher TB odds (adjusted OR [aOR] 12.90, 95% CI 8.84–18.82) compared to CD4+ >500 cells/μL. Cholesterol <3.9 mmol/L had higher TB odds (aOR 3.11, 95% CI 1.94–4.98) compared to cholesterol >5.5 mmol/L.

**Conclusion:**

In this Asia-Pacific cohort of adults living with HIV, low CD4+ cell count and low total serum cholesterol were associated with increased TB odds. Cholesterol may represent a low-cost adjunct marker to support TB risk stratification in PLHIV in endemic settings, but requires validation and evaluation of feasibility and cost-effectiveness.

## INTRODUCTION

Tuberculosis (TB), caused by the bacteria *Mycobacterium tuberculosis*, is the most frequent opportunistic infection among people living with HIV (PLHIV) and is responsible for over a quarter of all HIV-associated deaths ^1,2^. Nearly half of the HIV-associated deaths are estimated to be associated with undiagnosed TB (45.8%) ^3^. This failure to detect TB contributes significantly to mortality in PLHIV. The World Health Organization (WHO) estimates that just ten countries account for the majority of undiagnosed cases worldwide; eight of these are located in the Southeast Asian region ^1^. Although CD4+ cell depletion is a well-established risk factor for TB in HIV-positive individuals, the identification of additional routine clinical markers associated with TB diagnosis could support more effective clinical screening strategies in high-burden settings.

Previous analysis of clinical trial data from the SECONDLINE study ^4^, which enrolled participants from sites across Africa, Asia, and South America, identified that the routine laboratory markers, cholesterol and platelets, together with CD4+ cell counts, were inversely associated with the risk of incident TB in PLHIV receiving second-line antiretrovial (ART) treatment ^5^. Researchers have established the link between low CD4+ cell counts and TB, leading to the routine inclusion of CD4 monitoring in clinical care for PLHIV in TB-endemic settings ^6,7^. There is also a growing body of research, principally in the general population, reporting low total cholesterol as a clinical finding at diagnosis of *M. tuberculosis* infection and associated with a greater risk of TB disease, independent of BMI ^8–12^. The evidence for the role of platelets in TB disease is variable. While elevated platelet counts are often a sign of inflammation associated with TB disease severity ^13^, low platelet counts have been identified as a presenting sign of TB in endemic areas ^14^. There is limited data on the relationship of these clinical markers to TB disease in PLHIV in the Asia-Pacific region. This study uses data from a large, multinational cohort in the Asia-Pacific region, to investigate associations between TB diagnoses and serum cholesterol, platelet counts, and CD4+ cell counts.

## METHODS

### Study Design and Population

We conducted a cross-sectional study among adults living with HIV enrolled in the TREAT Asia HIV Observational Database (TAHOD), a regional cohort of the International Epidemiology Databases to Evaluate AIDS (IeDEA) Asia-Pacific collaboration. TAHOD is a collaborative observational cohort study of adults living with HIV in the Asia-Pacific region [15], which collected routine clinical data from PLHIV enrolled at clinical sites between 2003 and 2021. Observational data evaluated in this study come from 20 TAHOD sites in 12 Asia-Pacific countries: Cambodia (n = 1 site), Hong Kong SAR, China (1), India (3), Indonesia (2), Japan (1), Malaysia (2), Philippines (1), Singapore (1), South Korea (1), Taiwan (1), Thailand (4), and Vietnam (2).

We used a matched case-control design to compare people with and without TB diagnoses under similar clinical conditions. Matching on calendar time and site to control for variation in TB diagnostic and treatment practices across countries and over time ^15^.

### Definition of TB

TB was any *M. tuberculosis* infection, either definitive or presumptive. A definitive diagnosis included a confirmed culture of *M. tuberculosis* complex from a clinical specimen. A presumptive diagnosis included a demonstration of acid-fast bacilli in a clinical specimen, histopathology of a person with clinical signs or symptoms compatible with TB disease, or a successful response to therapy for TB with two or more anti-TB medications.

### Case and Control Selection

**FIGURE 1.**
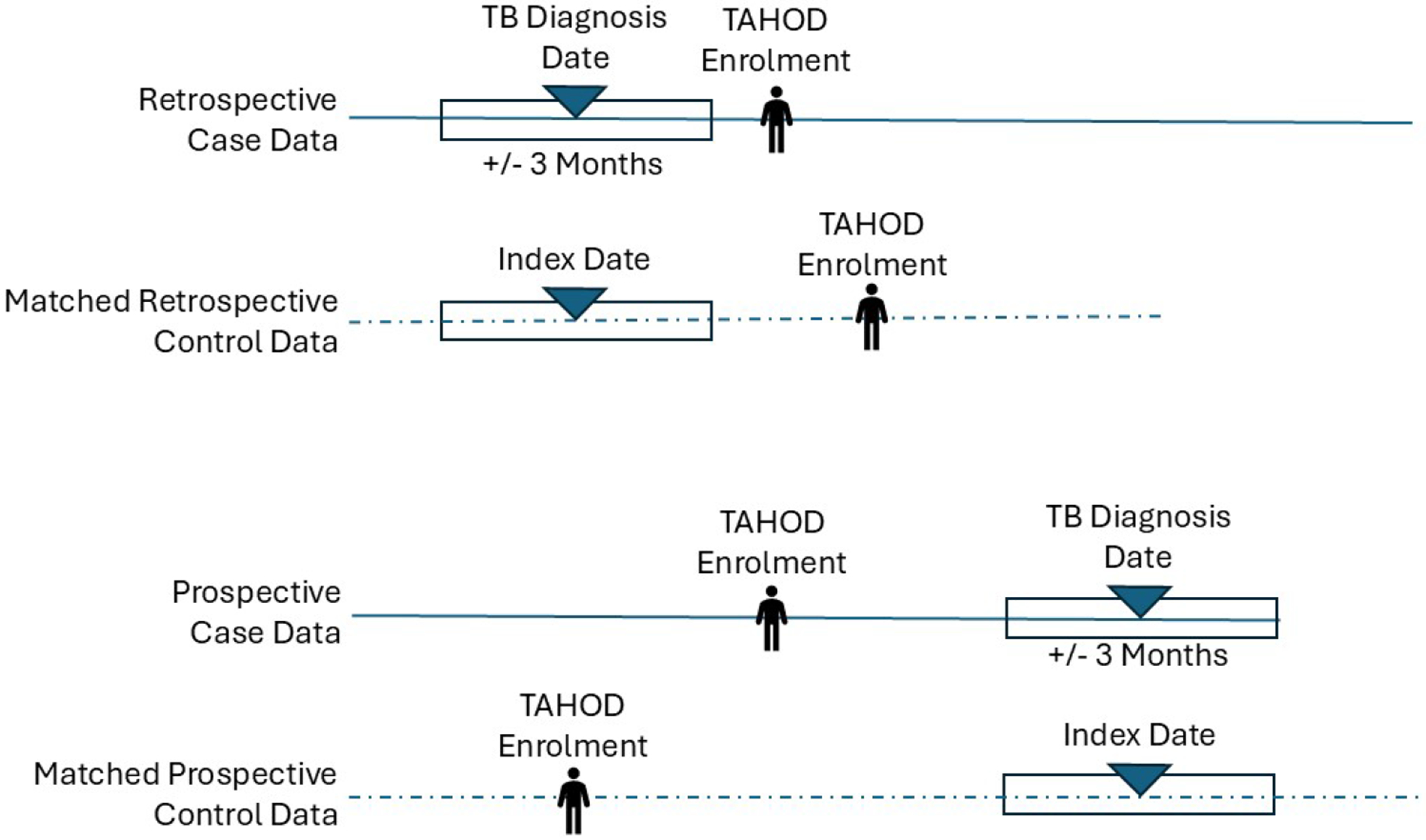
Illustration of the ±3-month data inclusion window used to define covariate observations for TB cases and matched controls. For each case, the window was centered on the TB diagnosis date. Matched control data were selected from the same calendar period, aligned with the case’s window, to ensure comparable timing of clinical and laboratory measurements.

Cases were participants diagnosed with TB at any time, classified as either prospective (diagnosed after TAHOD enrollment) or retrospective (diagnosed before cohort entry). Only the first TB episode was included, and individuals diagnosed before age 18 were excluded. For each cases, we used clinical and laboratory data collected within ±3 months of their first TB diagnosis date (Fig. 1). Cases were randomized before matching to minimize selection bias.

Each case was matched to two controls by clinical site and calendar time, using a ±3-month window around the case TB diagnosis date (Fig. 1). Eligible controls had no recorded TB diagnosis and had data available during the same time window at the same clinic. Prospective controls were required to have available data during the same calendar period as the matched prospective case’s TB diagnosis. Retrospective controls were required to have data within the window period of the matched retrospective case. When more than two controls were available, we randomly sampled two controls from eligible controls. If two or fewer potential controls were available, all were selected. Selected controls were then removed from the pool to prevent resampling.

### Covariates

Primary covariates of interest were total serum cholesterol, platelet count, and CD4+ cell count. Additional covariates included sex, age, body mass index (BMI), smoking status (never/ever), hepatitis B or C co-infection, HIV exposure category, ART regimen, prior AIDS diagnosis, and HIV viral load. Hepatitis B or C co-infection was established using reported serological and virological assay results. People with a positive HBsAg or anti-HBc result were identified as having HBV co-infection, and a positive HCV antibody or detectable RNA result identified HCV co-infection. BMI was calculated using weight measurements collected within the ±3-month window (Fig. 1), assuming stable adult height. Continuous variables were categorized using established clinical thresholds: total serum cholesterol (<3.9, 3.9 – 5.5, >5.5 mmol/L) ^16^; platelet counts (<150 and ≥150 x 10^9^/L) ^17^; CD4+ cell count (<200, 200 - 500, >500 cells/μL) ^18^; HIV viral load (≤1000 copies/mL and >1000 copies/mL) ^18^; and BMI (<18.5, >=18.5 - <23, >23 kg/m^2^) ^19^. The clinical site and calendar time were accounted for through matching, and country income level was included for descriptive purposes only.

When multiple clinical or laboratory measurements were available within the defined ±3-month timeframe (Fig. 1), we selected the observation closest to the date of the case TB diagnosis for matched case-controls. Each participant contributed a single observation for each covariate.

### Statistical Analysis

Associations between covariates and TB diagnosis were assessed using conditional logistic regression, accounting for the matched design. Unreported values were coded as a separate indicator category for each covariate ^20^. Global p-values for covariates were generated using Wald tests, with the unreported category excluded, to assess heterogeneity and test for overall association. Multivariable models were analyzed using backward stepwise selection, including covariates with P < 0.10 in univariate analysis and retaining those with a global P < 0.05 in the final model. Data management, matching, and analyses were conducted using SAS version 9.4 (SAS Institute Inc., Cary, NC, USA), R version 4.4.2 (R Foundation for Statistical Computing, Vienna, Austria), and STATA version 18 (Stata Corp., College Station, TX, USA).

### Ethical Considerations

All participating sites had ethics approvals from local ethics committees, the data management and biostatistical center (UNSW Sydney Ethics Committee), and the coordinating center (TREAT Asia/amfAR).

## RESULTS

### Demographic and Clinical Characteristics of the Cohort

We included 4,244 PLHIV from 20 sites: 1,427 TB cases and 2,817 controls. Where possible, we matched TB cases with two controls; however, we did include 37 TB cases with only one eligible control matched. TB diagnoses included in the analysis occurred between 1996 and 2022. Among the 1,427 TB cases, 296 (20.74%) were prospective cases (diagnosed after cohort entry), and 1,131 (79.26%) were retrospective cases (diagnosed before cohort enrolment).

Table 1 summarizes the characteristics of cases and controls. At the time of TB diagnosis, the median age of cases was 35 years (interquartile range [IQR] 30–42), and 75.26% were male. Of 1,427 TB cases, 678 (47.51%) were from lower-middle-income countries, 567 (39.73%) from upper-middle-income countries, and 182 (12.75%) from high-income countries. Over half of the cases (962 [67.41%]) occurred in countries with a high TB burden ^1^. Of the 1,228 (88.57%) TB cases with available CD4+ cell counts, 918 (64.33%) had CD4+ cell counts below 200 cells/μL. Among 515 (36.09%) TB cases with reported cholesterol data, 260 (18.22%) had total cholesterol levels below 3.9 mmol/L. Platelet data were scarce; of the 105 (8.46%) TB cases with platelet measurements available, only 10 (0.70%) had platelet levels below 150 × 10^9^/L.

### Factors associated with TB diagnosis

Table 2 presents the factors associated with a diagnosis of TB. Variables with p < 0.10 in univariate analysis included sex, age, smoking status, BMI, hepatitis C co-infection, HIV exposure mode, ART combination, prior AIDS diagnosis, HIV viral load, CD4+ cell count, and total cholesterol.

In the multivariable model, female sex was associated with lower odds of TB diagnosis (adjusted OR [aOR] 0.52, 95% CI 0.44–0.63) compared to male sex. Compared to normal BMI (>=18.5 and <23), low BMI (<18.5) had higher odds of TB diagnosis (aOR 2.36, 95% CI 1.88 – 2.96), while high BMI (>23) had lower odds of TB (aOR 0.44, 95% CI 0.33 – 0.58). Prior AIDS diagnosis (aOR 2.14, 95% CI 1.74 - 2.65) and HIV viral load >1000 copies/mL (aOR 3.15, 95% CI 2.21 - 4.49) were associated with higher odds of TB diagnosis. CD4+ cell counts showed an inverse association with the diagnosis of TB. Compared to high CD4+ cell counts (>500 cells/μL), <200 cells/μL (aOR 12.90, 95% CI 8.84–18.82) and 200–500 cells/μL (aOR 3.08, 95% CI 2.12–4.48) were associated with higher odds of TB diagnosis. Total cholesterol was also inversely associated with TB. Compared to high cholesterol (> 5.5 mmol/L), low cholesterol (<3.9 mmol/L) was associated with higher odds of TB (aOR 3.11, 95% CI 1.94 – 4.98), and cholesterol between 3.9–5.5 mmol/L also showed increased odds of TB (aOR 1.99, 95% CI 1.28–3.11). No association was observed between platelet counts and the diagnosis of TB.

## DISCUSSION

In this analysis of TB cases in a cohort of adults living with HIV in the Asia-Pacific, we found that low CD4+ cell counts (<200 cells/μL) and low total serum cholesterol (≤3.9 mmol/L), adjusted for sex, BMI, prior AIDS, and HIV viral load were associated with greater odds of TB diagnosis. The observed associations of sex and BMI with TB diagnosis in this analysis are consistent with established epidemiologic patterns, with adult males having a higher likelihood of TB ^1^, and BMI associated with TB ^21^. By leveraging TAHOD’s nearly two decades of routine clinical data from PLHIV in TB-endemic countries, this analysis provides insight into a real-world population beyond controlled trial settings ^22^.

Our results in the population of PLHIV align with the growing evidence that low cholesterol is associated with TB. Similar observations in SECONDLINE participants found an association between low baseline cholesterol and subsequent TB in PLHIV receiving antiretroviral therapy (ART) ^5^. However, such findings in HIV-positive populations remain limited, as research on dyslipidemia and TB frequently excludes PLHIV, likely to avoid confounding by HIV co-infection, a well-known risk factor for TB ^1,23^. While the mechanisms underlying low cholesterol and TB are poorly understood, observational studies in the general population have consistently found an association between low serum cholesterol and TB, independent of BMI ^10,11^. Many studies assess serum cholesterol after diagnosis, comparing TB cases with healthy controls ^5,8,9^. More recently, longitudinal analyses have strengthened the link between cholesterol and TB by showing that cholesterol levels serve as predictors of future TB risk. A study of the South Korean National Health Database found that low total cholesterol (<4.5 mmol/L) significantly increased the risk of developing TB; notably, in this study, HIV status was not reported ^12^. Existing research suggests that the cholesterol-TB association may be even stronger among PLHIV. For example, in an Ethiopian cohort, serum cholesterol levels were significantly lower in TB patients co-infected with HIV compared to HIV-negative counterparts ^8^.

While this study can not establish causality, potential mechanisms linking cholesterol and TB warrant further exploration. Cholesterol appears to play a complex role in both host immunity and the pathogenesis of *M. tuberculo*sis. Experimental evidence suggests that cholesterol levels have a direct influence on immune competence. *In vitro* studies have demonstrated that when cholesterol availability is restricted, T cell function is impaired, including reduced T cell proliferation and impaired antigen presentation in mouse models ^24^. Suggesting that low serum cholesterol may be either a proxy for or a driver of immunocompromise, increasing susceptibility to TB. This relationship is complicated by evidence that *M. tuberculosis* itself requires host cholesterol to establish and maintain infection. Initially, bacteria use host-derived cholesterol to facilitate entry into macrophages ^25,26^. Once established, *M. tuberculosis* manipulates host lipid metabolism by enhancing cholesterol uptake and suppressing efflux, maintaining intracellular cholesterol availability for energy production, and facilitating chronic infection ^27,28^. In this context, TB disease may lower cholesterol levels by directing the host’s lipid resources to infected cells and disrupting normal cholesterol balance. Understanding these competing roles of cholesterol, both as a host defense mechanism and as a resource for pathogens, is important for interpreting cholesterol’s clinical significance in assessing TB risk. Future well-designed prospective cohort studies are warranted to establish the causal relationship between cholesterol levels and the risk of TB.

HIV co-infection is a known risk factor for TB disease ^29^. The association of low CD4+ cell counts, high viral load, and increased odds of TB diagnosis in this analysis is consistent with the established evidence of HIV-associated TB ^30^. We examined whether cholesterol remained associated with TB in the group of individuals with well-controlled HIV. Among individuals with suppressed HIV viral loads (≤1000 copies/mL), there were higher proportions of TB diagnosis among those with low cholesterol compared to high cholesterol (34.6% vs. 8.9%, p = 0.011). This finding suggests that the association between cholesterol and TB remains relevant even in patients with effective HIV treatment. These findings are particularly relevant given the evolving lipid landscape in HIV care. Prior to ART availability, HIV infection commonly caused low cholesterol levels ^31^. Following ART initiation, total cholesterol levels are observed to return to pre-infection serum levels in PLHIV over time ^32^. However, with the introduction of newer antiretroviral agents, cholesterol levels are increasing, and PLHIV are now at greater risk of metabolic syndrome ^33,34^. Consequently, current research on lipids in PLHIV has primarily focused on the link with non-communicable diseases ^35,36^. Given the increased cardiovascular risk in PLHIV, lipid management has become standard clinical practice, primarily through statin therapy ^37,38^. This distinction between pathological and therapeutic cholesterol reduction is supported by the previously mentioned Korean study ^12^. While low cholesterol levels were associated with increased TB risk, statin use appeared to modify this relationship, with the association losing statistical significance among statin users. Since the evidence indicates that therapeutic lowering of cholesterol does not increase TB susceptibility, standard lipid management practices for PLHIV with elevated cholesterol need not be altered due to TB concerns.

The observed association between low cholesterol and TB in PLHIV raises the possibility that cholesterol monitoring may serve as a potential TB screening tool. In resource-limited settings where CD4+ testing is no longer routine ^39^, cholesterol testing might offer an alternative approach to TB risk stratification, particularly if it proves more accessible than immunological monitoring. However, implementation needs to consider the specific resource constraints of each setting. Health economic evaluations would be essential to determine cost-effectiveness and clinical utility compared to existing screening approaches. The optimal target population and integration with existing clinical workflows would require careful evaluation before any implementation.

These results have some important limitations. First, the observational design of the cohort data may limit the generalizability. TAHOD participants were selected based on their likelihood of remaining in care, which may bias toward those already engaged with healthcare. Due to the relatively small number of prospective TB cases, we included retrospective cases to increase statistical power. However, as pre-enrolment data were submitted voluntarily, historical TB cases may be underreported. While including retrospectively identified cases may introduce bias, we matched the retrospective cases with retrospective controls during the same calendar time, ensuring comparisons were made within the same snapshot period. Given the relatively low incidence of TB in the cohort, including retrospective cases allowed us to maximize the sample size. Data completeness represents another limitation. Cholesterol measurements were available for 1,761 participants (41.49% of the study population), while platelet counts were available for only 312 participants (7.35%). We used the missing indicator method to retain matched pairs with incomplete covariate data, preserve the matching structure, and maximize the sample size. While this method reduces standard error, residual bias due to missingness cannot be ruled out ^20^. Besides the limitations of quantitative data, inconsistencies in clinical classification also restricted our analysis. TAHOD sites inconsistently reported the anatomical site and severity of TB disease, particularly pulmonary versus extrapulmonary TB classification. As a result, we were unable to assess risk factors according to anatomical site of TB. Additionally, the absence of data on TB prophylaxis, treatment regimens, and clinical outcomes prevented evaluation of interventions or treatment outcomes. Despite these limitations, this analysis provides crucial preliminary evidence that should be examined through well-designed prospective studies.

Several future research directions emerge from these findings. To better understand the underlying mechanisms, laboratory and longitudinal clinical studies are needed to clarify the biological pathways linking cholesterol and TB. Future research should assess whether cholesterol monitoring can improve TB risk assessment in routine HIV care in TB-endemic settings and evaluate the feasibility and clinical utility of such monitoring. In parallel, further investigation into the impact of statin therapy on TB risk among PLHIV is needed, given its potential dual role in lipid management and immune modulation. Finally, given the inconsistent findings in the literature, additional research is needed to clarify the role of platelet levels in TB development and whether platelet counts should be considered alongside cholesterol in risk assessment strategies.

### Conclusion

Low CD4+ cell count and low total serum cholesterol were associated with increased odds of TB diagnosis in this Asia-Pacific cohort of PLHIV. This analysis provides real-world evidence supporting the growing body of literature linking low cholesterol to TB across diverse populations. Further prospective research is needed to clarify underlying mechanisms direction of this association, and the potential clinical utility of cholesterol measurement in high-burden settings.

## Figures and Tables

**TABLE 1:** STUDY POPULATION DEMOGRAPHICS Primary covariates were total cholesterol, platelets, and CD4 count. Continuous variables were categorized using clinical thresholds. Missing values were included as an indicator category of the variable. Country income group was reported descriptively.

	Total (%)	Non-TB Controls (%)	TB Cases (%)
	4244 (100%)	2817 (66.38)	1427 (33.62)

Sex			
Male	2814 (66.31)	1740 (61.77)	1074 (75.26)
Female	1430 (33.69)	1077 (38.23)	353 (24.74)

Age (years)	*median* 36.00 [IQR: 30.00, 42.00]	*median* 36.00 [IQR: 30.00, 43.00]	*median* 35.00 [IQR: 30.00, 42.00]
<= 30	1131 (26.67)	766 (27.23)	365 (25.58)
31 - 40	1821 (42.95)	1169 (41.56)	652 (45.69)
41 - 50	889 (20.97)	1169 (41.56)	290 (20.32)
51 +	399 (9.41)	279 (9.92)	120 (8.41)

World Bank Income Classification			
Lower Middle	2018 (47.55)	1340 (47.57)	678 (47.51)
Upper Middle	1684 (39.68)	1117 (39.65)	567 (39.73)
High	542 (12.77)	360 (12.78)	182 (12.75)

Smoking			
Never Smoked	1649 (38.85)	1176 (41.75)	473 (33.15)
Ever Smoker	1325 (31.22)	827 (29.36)	498 (34.90)
Not Reported	1270 (29.92)	814 (28.90)	456 (31.96)

BMI (kg/m^2^)	median = 20.72 [IQR: 18.61, 23.15]	median = 21.48 [IQR: 19.39, 23.90]	median = 19.23 [IQR: 17.30, 21.36]
< 18.5	772 (18.19)	344 (12.21)	428 (29.99)
>= 18.5 and < 23	1566 (36.90)	1069 (37.95)	497 (34.83)
>= 23	851 (20.05)	708 (25.13)	143 (10.02)
Not Reported	1055 (24.86)	696 (24.71)	359 (25.16)

Hepatitis B Co-Infection			
Negative	2502 (58.95)	1660 (58.93)	842 (59.00)
Positive	724 (17.06)	484 (17.18)	240 (16.82)
Not Tested	1018 (23.99)	673 (23.89)	345 (24.18)

Hepatitis C Co-Infection			
Negative	2760 (65.03)	1863 (66.13)	897 (62.86)
Positive	466 (10.98)	281 (9.98)	185 (12.96)
Not Tested	1018 (23.99)	673 (23.89)	345 (24.18)

Mode of HIV Exposure			
Heterosexual	3111 (73.30)	2096 (74.41)	1015 (71.13)
MSM	519 (12.23)	357 (12.67)	162 (11.35)
IDU	609 (14.35)	360 (12.78)	249 (17.45)
Other/Unknown	5 (0.12)	4 (0.14)	1 (0.07)

ARV Treatment Regimen			
NNRTI+NRTI-based	2812 (66.26)	1841 (65.35)	971 (68.04)
PI-based	457 (10.77)	330 (11.71)	127 (8.90)
INSTI-based	52 (1.23)	28 (0.99)	24 (1.68)
Other	105 (2.47)	75 (2.66)	30 (2.10)
No ART	818 (19.27)	543 (19.28)	275 (19.27)

Previous AIDS Diagnosis Reported			
Yes	1260 (29.69)	1004 (35.64)	256 (17.94)
No	2984 (70.31)	1813 (64.36)	1171 (82.06)

HIV viral load (copies/mL)	median = 422.00 [IQR: 49.00, 92200.75]	median = 76.50 [IQR: 40.00, 14588.50]	median = 98853.50 [IQR: 1201.00, 334220.50]
<= 1,000	874 (20.59)	746 (26.48)	128 (8.97)
> 1,000	764 (18.00)	366 (12.99)	398 (27.89)
Not Reported	2606 (61.40)	1705 (60.53)	901 (63.14)

CD4 Cell Count (cells/mm^3^)	median = 225.00 [IQR: 80.00, 408.00]	median = 304.50 [IQR: 161.00, 483.00]	median = 83.50 [IQR: 33.00, 202.25]
< 200	1703 (40.13)	785 (27.87)	918 (64.33)
200 - 500	1369 (32.26)	1109 (39.37)	260 (18.22)
> 500	620 (14.61)	570 (20.23)	50 (3.50)
Not Reported	552 (13.01)	353 (12.53)	199 (13.95)

Platelets x 10^9^/L	median = 248.00 [IQR: 207.75, 315.75]	median = 244.00 [IQR: 207.00, 288.50]	median = 280.00 [IQR: 210.00, 361.00]
< 150	22 (0.52)	12 (0.43)	10 (0.70)
>= 150	290 (6.83)	195 (6.92)	95 (6.66)
Not Reported	3932 (92.65)	2610 (92.65)	1322 (92.64)

Total cholesterol (mmol/L)	median = 4.40 [IQR: 3.60, 5.30]	median = 4.70 [IQR: 3.80, 5.60]	median = 3.80 [IQR: 3.15, 4.60]
< 3.9	581 (13.69)	321 (11.40)	260 (18.22)
3.9 - 5.5	818 (19.27)	606 (21.51)	212 (14.86)
> 5.5	362 (8.53)	319 (11.32)	43 (3.01)
Not Reported	2483 (58.51)	1571 (55.77)	912 (63.91)

**TABLE 2:** CONDITIONAL LOGISTIC REGRESSION OF COVARIATES ASSOCIATED WITH TB DIAGNOSIS Models were stratified by matched sets. Global p-values were derived from post-estimation Wald tests. Multivariable models used backward stepwise selection (p < 0.10 entry; p < 0.05 retention).

			Univariate			Multivariate		
	Non-TB Controls (n =)	TB Cases (n =)	OR	95% CI	p	OR	95% CI	p
**Total**	2817	1427						

Sex								
Male	1740	1074	1			1		
Female	1077	353	0.51	0.44 - 0.59	<0.0001	0.52	0.44 - 0.63	<0.0001

Age (years)					0.0531			
<= 30	766	365	1					
31 - 40	1169	652	1.19	1.01 - 1.40	0.037			
41 - 50	599	290	1.03	0.85 - 1.26	0.748			
51 +	279	120	0.91	0.71 - 1.18	0.487			

Smoking					<0.0001			
Never Smoked	1176	473	1					
Ever/Current Smoker	827	498	1.54	1.31 - 1.81	<0.0001			
Not Reported	814	456	*1.47*	*1.23 - 1.75*	*<0.0001*			

BMI					<0.0001			<0.0001
< 18.5	344	428	2.77	2.30 - 3.34	<0.0001	2.36	1.88 - 2.96	<0.0001
>= 18.5 and < 23	1069	497	1			1		
>= 23	708	143	0.41	0.33 - 0.51	<0.0001	0.44	0.33 - 0.58	<0.0001
Not Reported	696	359	*1.05*	*0.83 - 1.33*	*0.679*	*1.03*	*0.77 - 1.37*	*0.846*

Hepatitis B Co-Infection					0.8339			
Negative	1660	842	1					
Positive	484	240	0.98	0.81 - 1.19	0.834			
Not Tested	673	345	*1.01*	*0.82 - 1.25*	*0.897*			

Hepatitis C Co-Infection					0.0010			
Negative	1863	897	1					
Positive	281	185	1.46	1.17 - 1.84	0.001			
Not Tested	673	345	*1.08*	*0.87 - 1.33*	*0.501*			

Mode of HIV Exposure					0.0002			
Heterosexual	2096	1015	1					
MSM	357	162	0.93	0.72 - 1.20	0.557			
IDU	360	249	1.54	1.25 - 1.89	<0.0001			
Other/Unknown	4	1	0.52	0.06 - 4.65	0.558			

ARV Treatment Regimen					0.0009			
NNRTI+NRTI-based	1841	971	1					
PI-based	330	127	0.65	0.51 - 0.84	0.001			
INSTI-based	28	24	2.15	1.05 - 4.44	0.037			
Other	75	30	0.63	0.39 - 1.02	0.060			
No ART	543	275	0.89	0.74 - 1.07	0.221			

Prior AIDS Diagnosis								
Yes	1004	256	2.83	2.39 - 3.36	<0.0001	2.14	1.74 - 2.65	<0.0001
No	1813	1171	1			1		

HIV viral load (copies/mL)					<0.0001			<0.0001
<= 1,000	746	128	1			1		
> 1,000	366	398	8.94	6.72 - 11.88	<0.0001	3.15	2.21 - 4.49	<0.0001
Not Reported	1705	901	*4.36*	*3.31 - 5.75*	*<0.0001*	*2.19*	*1.55 - 3.10*	*<0.0001*

CD4 Cell Count (cells/mm^3^)					<0.0001			<0.0001
< 200	785	918	20.11	14.21 – 28.47	<0.0001	12.90	8.84 – 18.82	<0.0001
200 - 500	1,109	260	3.19	2.22 - 4.48	<0.0001	3.08	2.12 – 4.48	<0.0001
> 500	570	50	1			1		
Not Reported	353	199	*9.03*	*6.13 - 13.31*	*<0.0001*	*7.95*	*5.19 - 12.18*	*<0.0001*

Platelets x 10^9^/L					0.2229			
< 150	12	10	1.74	0.71 - 4.27	0.223			
>= 150	195	95	1					
Not Reported	2610	1322	*1.03*	*0. 71 - 1.49*	*0.876*			

Total cholesterol (mmol/L)					<0.0001			<0.0001
< 3.9	321	260	7.74	5.23 - 11.44	<0.0001	3.11	1.94 – 4.98	<0.0001
3.9 - 5.5	606	212	2.95	2.04 – 4.28	<0.0001	1.99	1.28 – 3.11	0.002
> 5.5	319	43	1			1		
Not Reported	1571	912	*1.94*	*1.59 - 2.37*	*<0.0001*	*3.52*	*2.29 – 5.43*	*<0.0001*

## TAHOD study members.

V Khol, V Ouk, C Pov, V Bun, National Center for HIV/AIDS, Dermatology & STDs, Phnom Penh, Cambodia; MP Lee, PCK Li, TS Kwong, KY Yeung, Queen Elizabeth Hospital, Hong Kong SAR; N Kumarasamy, S Poongulali, B Faith, VHS-Infectious Diseases Medical Centre, Chennai Antiviral Research and Treatment Clinical Research Site (CART CRS), Voluntary Health Services, Chennai, India; S Pujari, K Joshi, S Gaikwad, A Chitalikar, Institute of Infectious Diseases, Pune, India; RT Borse, V Mave, I Marbaniang, S Nimkar, BJ Government Medical College and Sassoon General Hospital, Pune, India; IKA Somia, TP Merati, NM Dewi Dian Sukmawati, F Yuliana, Faculty of Medicine Udayana University - Ngoerah Hospital, Bali, Indonesia; E Yunihastuti, B Wicaksana, A Widhani, S Maria, Faculty of Medicine Universitas Indonesia - Dr. Cipto Mangunkusumo General Hospital, Jakarta, Indonesia; J Tanuma, H Gatanaga, H Uemura, Y Koizumi, National Center for Global Health and Medicine, Tokyo, Japan; JY Choi, JH Kim, JE Park, Division of Infectious Diseases, Department of Internal Medicine, Yonsei University College of Medicine, Seoul, South Korea; YM Gani, TK Heng, NA Misnan, SK Chidhambaram, Hospital Sungai Buloh, Sungai Buloh, Malaysia; I Azwa, A Kamarulzaman, SF Syed Omar, S Ponnampalavanar, University Malaya Medical Centre, Kuala Lumpur, Malaysia; RA Ditangco, MK Pasayan, JB Sornillo, Research Institute for Tropical Medicine, Muntinlupa City, Philippines; HP Chen, YJ Chan, PF Wu, Taipei Veterans General Hospital, Taipei, Taiwan; CY Choy, PH Lee, PA Kumar, Z Ferdous, National Centre for Infectious Diseases, Tan Tock Seng Hospital, Singapore; A Avihingsanon, N Hiranburana, C Wongvoranet, C Ruengpanyathip, HIV-NAT/Thai Red Cross AIDS and Infectious Diseases Research Centre, Bangkok, Thailand; S Kiertiburanakul, A Phuphuakrat, L Chumla, N Sanmeema, Faculty of Medicine Ramathibodi Hospital, Mahidol University, Bangkok, Thailand; R Chaiwarith, T Sirisanthana, J Praparattanapan, K Nuket, Faculty of Medicine and Research Institute for Health Sciences, Chiang Mai University, Chiang Mai, Thailand; S Khusuwan, P Kambua, S Pongprapass, J Limlertchareonwanit, Chiangrai Prachanukroh Hospital, Chiang Rai, Thailand; TN Pham, KV Nguyen, DTH Nguyen, DT Nguyen, National Hospital for Tropical Diseases, Hanoi, Vietnam; CD Do, AV Ngo, LT Nguyen, Bach Mai Hospital, Hanoi, Vietnam; AH Sohn, JL Ross, B Petersen, TREAT Asia, amfAR - The Foundation for AIDS Research, Bangkok, Thailand; K Petoumenos, A Jiamsakul, D Rupasinghe, The Kirby Institute, UNSW Sydney, NSW, Australia.
